# Human–Machine Interface: Multiclass Classification by Machine Learning on 1D EOG Signals for the Control of an Omnidirectional Robot

**DOI:** 10.3390/s21175882

**Published:** 2021-08-31

**Authors:** Francisco David Pérez-Reynoso, Liliam Rodríguez-Guerrero, Julio César Salgado-Ramírez, Rocío Ortega-Palacios

**Affiliations:** 1 Mechatronic Engineering, Universidad Politécnica de Pachuca (UPP), Zempoala 43830, Mexico; david_perez@upp.edu.mx; 2 Research Center on Technology of Information and Systems (CITIS), Electric and Control Academic Group, Universidad Autónoma del Estado de Hidalgo (UAEH), Pachuca de Soto 42039, Mexico; 3 Biomedical Engineering, Universidad Politécnica de Pachuca (UPP), Zempoala 43830, Mexico

**Keywords:** EOG, one hot encoding, machine learning, omnidirectional robot

## Abstract

People with severe disabilities require assistance to perform their routine activities; a Human–Machine Interface (HMI) will allow them to activate devices that respond according to their needs. In this work, an HMI based on electrooculography (EOG) is presented, the instrumentation is placed on portable glasses that have the task of acquiring both horizontal and vertical EOG signals. The registration of each eye movement is identified by a class and categorized using the one hot encoding technique to test precision and sensitivity of different machine learning classification algorithms capable of identifying new data from the eye registration; the algorithm allows to discriminate blinks in order not to disturb the acquisition of the eyeball position commands. The implementation of the classifier consists of the control of a three-wheeled omnidirectional robot to validate the response of the interface. This work proposes the classification of signals in real time and the customization of the interface, minimizing the user’s learning curve. Preliminary results showed that it is possible to generate trajectories to control an omnidirectional robot to implement in the future assistance system to control position through gaze orientation.

## 1. Introduction

The EOG signal is generated by the potential difference between the retina and the cornea of the eye by means of superficial electrodes; the horizontal (left–right) and vertical (up–down) eye movements can be detected [1,2,3]. In recent years, HMI has been implemented using EOG since its acquisition is less invasive compared to electroencephalography (EEG) [4,5,6]. In addition, artificial intelligence algorithms have been used which allow the classification of EOG signals for the control of wheelchairs, orthotics, assistance robots and HMI [7,8,9]. In [10], for example, the horizontal EOG channel is used to generate control commands for a lower limb orthosis, these commands are detected in a three-second sampling window to avoid false activations of the system and the processing is done in machine language. In [11], an Internet search engine was developed using horizontal and vertical EOG signals, the user’s impulses are obtained by deriving the signal and using a prediction algorithm of words, getting a response time of between 80 and 100 s. In [12], a hybrid brain–computer interface (hBCI) is carried out, through the union of EOG and EEG. Classification is done using the EEG signal with a Support Vector Machine (SVM) and the EOG signal is used to eliminate noise on EEG acquisition. In [13], an interface method is proposed to improve the letter selection on a virtual keyboard, where an EOG-guided mouse points to interactive buttons with audio; click is controlled by blinking.

Other systems classifying EOG signals using fuzzy logic and a database that store waveform information from different users have been developed [14,15,16,17,18] in order for the interface to compare the parameters of each user with previously established commands. One of the most representative works is presented in [19]; Fuzzy PD control is applied to the horizontal EOG channel that generates a wheelchair’s rotation to the right or left and the vertical EOG indicates forward or reverse. In [20], a writing system for people with disabilities is designed; the similarity of the trajectories generated by the movement of the eye and the shape of the letters is determined by fuzzy Gaussian membership functions. The training generates a database, which is the input to a multilayer neural network determining the letter that the user wants to write using EOG signals. In addition, the EOG has been applied in other fields such as industrial robotics; for example, in [21] a speed control system of a FANUC LR Mate 200iB industrial robot is developed using EOG. The signal amplitude is classified; the voltage value is previously divided into three thresholds; the user must reach the defined amplitude, otherwise the robot will not activate. The authors in [22] also developed a portable EOG acquisition system, which generates position control commands for an industrial robot using a nine-state machine, concerning which it was tested whether making the end effector followed five points; the obtained response time was of 220 s with trained users. In [23] a review of EOG based human–computer interface systems is presented; the work of 41 authors is explained, where the interfaces used to move a device always generate points in coordinates X-Y, as is the case for control of wheelchairs, Mohd et al [19]. In this paper, research did not generate a three-dimensional workspace; unlike the one presented in [24] where the EOG signals activate a robot with three degrees of freedom in 3D Cartesian space, the Cartesian coordinates X, Y, Z are generated by a fuzzy classifier that is automatically calibrated using optimization algorithms. The system response time, from the user’s eye movement until the robot reaches the desired position, is 118 s. This is less than that reported in the works presented in [23]; however, in this research it was found that to control a device that moves in a Cartesian space a state machine is insufficient to describe all the points in the workspace and does not allow track trajectories.

Some authors have as an alternative method the hybrid Brain–Computer Interfaces (BCIs) using eye-tracking to control robot models. Reference [25] presents the motion of an industrial robot controlled with eye movement and eye tracking via Ethernet. Reference [26] presents a hybrid wearable interface using eye movement and mental focus to control a quadcopter in three-dimensional space. Reference [27] developed a hybrid BCI to manipulate a Jaco robotic arm using natural gestures and biopotentials. Reference [28] presented a semi-autonomous hybrid brain–machine interface using human intracranial EEG, eye tracing and computer vision to control an upper limb prosthetic robot.

In regards to the EOG work related to classifiers, Fang et al. published in their paper advances about visual writing for Japanese Katakana [20]. Since Katakana is mainly composed of straight lines, researchers developed a system to recognize 12 basic types of hits. By recognizing these strokes, the proposed system was able to classify all Katakana characters (48 letters). For six participants, Katakana recognition accuracy was 93.8%. In this study, a distinguishing feature implemented was the continuous eye writing. By ignoring small eye movements, the system could recognize the eye writing of multiple letters continuously without discrete sessions. The average entry rate was 27.9 letters per min. Another work related to eye movement is [29]. There, character classifiers written by the eyes were implemented using an artificial neural network (quantification of learning vectors) for eye writing recognition. The average accuracy in character detection was 72.1%. In works of Fang and Tsai, eye movement is applied to writing; we use them to create complex trajectories of a robot’s movements; in addition, machine learning classifiers are used to analyze eye movement. Computational models were developed to identify antioxidants in the laboratory and machine learning was used for this purpose. The validation method used in this study is 10-fold cross validation, whereas in Fang and Tsai, the following validation metrics were used: Sensitivity of 81.5%, specificity of 85.1% and accuracy of 84.6%. In [30], the random forest classification algorithm is used to validate the efficiency of the computational method. Genes are the subject of study in computational biology and models of classification algorithms have been proposed to determine essential genes and sequencing problems. The metrics used for the validation method were: Sensitivity 60.2%, specificity 84.6%, accuracy 76.3%, area of Receiver Operating Characteristic (ROC) curves, also called AUC with a value of 0.814 [31]. The aforementioned study demonstrated the importance of supervised classification and the metrics used, metrics that are determinative and recognized by researchers in machine learning, are reliable metrics to measure the accuracy of classifiers.

Three contributions are presented in this work: First, the designed acquisition system allows to obtain the EOG signal, which is free from interference induced noise, by applying a digital filter which is tuned analyzing the EOG frequency spectrum in real time, for selecting its cutoff frequency; the second contribution is the verification of the performance of different classifiers to choose the best algorithm for the EOG signal model and to control a robotic system, based on the result of precision, accuracy and computational cost for the development of the model in an embedded system; the third contribution proposed is the discrimination of the involuntary potentials model such as blinking; this characteristic does not affect the operation of the classifier, taking this property as a total stop of the system. The assistance system implements modeling through a Multilayer Neural Network (MNN) to generalize the classification of EOG signals. So, if there is an amplitude variation of the signal due to user change or clinical problems, the algorithm must search the dataset for an entry for the classifier and thus assign a response to the system. The system presented in this work customizes the classification system and adapts to the individual properties of the user.

Section 2.1 describes in detail each of the classifiers implemented to choose the best one for identifying the eye registration of both EOG channels and introduces the basics of the EOG signal. Section 2.3 details the design of the HMI. In Section 2.4 using machine learning and the horizontal and vertical EOG signal, the Cartesian coordinates are generated to position a robot using a PID control. In Section 3 a test is presented to evaluate the response time of the proposed system and a discussion of the contributions of the developed interface is made.

## 2. Materials and Methods

### 2.1. Classifiers

#### 2.1.1. Multilayer Perceptron (MLP)

MLP is a neural network that aims to solve classification problems when classes cannot be separated linearly. This neural network mainly consists of three types of layers which are the input layer, the intermediate or hidden layers and the output layer [32]. Researchers in machine learning consider this classifier to be a good pattern classifier. The classifier works as follows: Neurons whose output values belong to the corresponding class are in the output layer. Neurons in the hidden layer, as a propagation rule, use the weighted sum of the inputs with the synaptic weights and a sigmoid transfer function is applied to this sum. The backpropagation error uses the root mean square error as a cost function.

#### 2.1.2. Tree-Type Classifiers

There are tree-type classifiers such as C4.5, ID3, random forest and random tree and J48 [33,34]. These decision tree algorithms can be explained as follows: For iteration *n* and taking as a criterion an already established variable, the predictor variable is searched to decide the cut that was made as well as the exact cut point where the mistake made is minor. This would happen when the confidence levels are higher than those established. After the cutoff, the algorithm will execute if the predictor variables are above the defined higher confidence level. The level of confidence is important since given too many subjects and variables, the tree will result in a large one. To avoid this situation, the size of the tree is limited by assigning a minimum number of instances per node. These algorithms are the most used in the classification of patterns.

#### 2.1.3. Naïve Bayes (NB)

Naïve Bayes classifier is widely used in machine learning. It is based on Bayes’ theorem [35]. Bayes proposed that we learn from the world by approximations and that the world is neither probabilistic nor uncertain, which allows us to get very close to the truth the more evidence there is. This classifier assumes that the presence or absence of an attribute is not probabilistically related to the presence or absence of other attributes, different from what happens in the real world. The Naïve Bayes classifier consists of converting the data set into a frequency table. In addition, a probability table is created for the various events to occur. Naïve Bayes is applied to calculate the posterior probability of each class and the prediction class is the class with the highest probability. The classifier, due to its simplicity, allows to easily build probability-based models with very good performance.

#### 2.1.4. The K Nearest Neighbors (K-NN)

The K-Nearest Neighbor (K-NN) classifier is a widely used algorithm in supervised learning [36]. The concept of the classifier is intuitive. Each new attribute that is presented to the K-NN is classified to the class of its closest neighbor. The algorithm calculates the distance of the new attribute with respect to each of the existing attributes, the distances are ordered from least to greatest and the class with the highest frequency and the shortest distance is selected [37,38,39,40].

#### 2.1.5. Logistic Classifier (Logistic)

This classifier is based on logistic regression [41]. Logistic regression, because it does not require many computing resources, is widely used in machine learning as it turns out to be very efficient. The most common models of logistic regression are the classification of a binary value (yes or no; true or false) and the logistic regression model is the multinomial (more than two possible outcomes). The Logistic classifier, to classify or predict, assigns actual values based on the probability that the input belongs to an existing class. Probability is calculated using a sigmoid function, where the exponential function plays a very important role.

#### 2.1.6. Support Vector Machines (SVM)

The concept of SVM is based on finding the hyperplane to separate the classes in the data space [42,43,44]. This algorithm is born from the theory of statistical learning. Optimization of analytical functions serves as the basis for the design and operation of SVM algorithms.

#### 2.1.7. Performance Measures

Within the supervised classification there are two processes or phases; one phase is the learning phase and the other phase is the classification phase [45]. A classifier should always have one data set for the training phase (P_train), which is also called a training class and another data set for testing the performance of the class, which is called a test class (P_test). Once the classifier learns, it is presented with a test class and as a result the presented pattern sets will be assigned to the corresponding classes. Patterns will not always be classified correctly, indicating that this is acceptable according to the no free lunch theorem [46,47].

As the data acquired is stored in a set of data or attributes, a partition of the total data set must be performed through a validation method. The method used in this paper is the cross-validation method. This method guarantees that the classes are distributed proportionally in each fold. The cross-validation method consists of dividing the total data set into k folds. k must be a positive integer and the most used values for k in the state of the art are k = 5 and k = 10 [48,49]. For this paper, the cross-validation method used will be k = 10.

**Figure 1:**
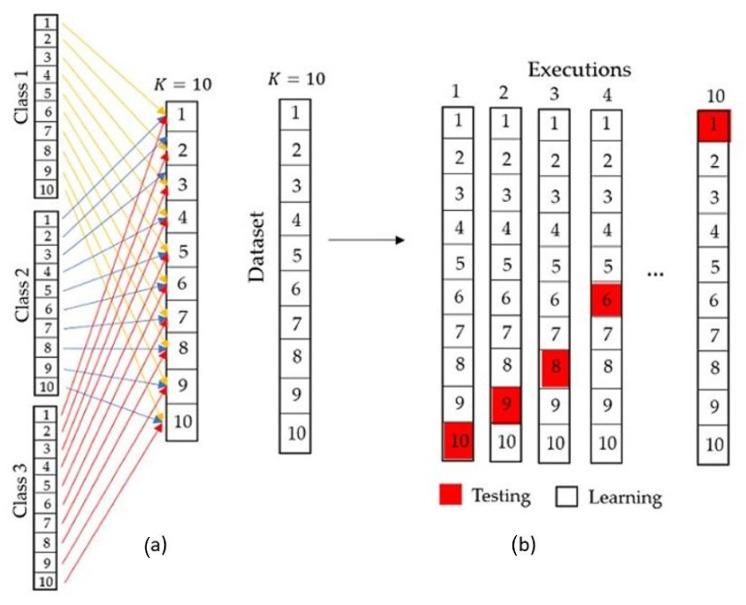
(**a**) The 10-fold stratified cross validation method. A data set is divided into three classes and located into 10 folds. (**b**) Operation of the 10-fold stratified cross validation method. The process consists of performing 10 iterations; in the first iteration, the classifier learns with the first 9 folds and the last is used for testing; the second leaves fold 9 to test and learn with the remaining folds and so forth.

Figure 1 exemplifies the behavior of the 10-fold cross-validation method, a data set divided into three classes located into 10 folds is shown schematically. The process to create the 10-fold cross-validation consists of taking the first pattern of class 1 and placing it in the 1 fold; the same is done for the second pattern, albeit placed in the 2 folds. This process is repeated until Pattern 10 from Classes 1–3 are placed on the 10 fold. The process of 10-fold cross-validation consists of performing 10 iterations. In the first iteration, the classifier learns with the first 9 folds and the last fold is used for testing. The second iteration leaves fold 9 to test and learn with the remaining folds and this process is repeated 10 times as shown in Figure 1b.

**Figure 2:**
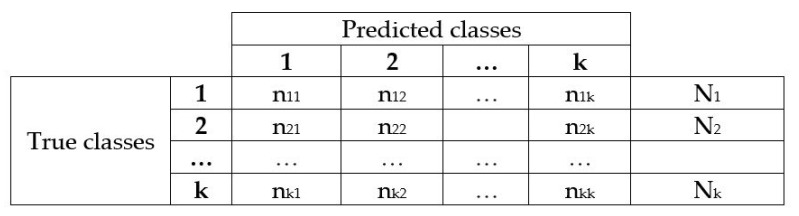
Confusion matrix for k classes.

According to the confusion matrix in Figure 2, it is established that the *i*-th letter (1≤i≤k) allows defining the three performance measures in the confusion matrix, which are sensitivity, specificity and balanced accuracy [50,51], measurements used in this paper.

$N_{i}$ indicates the total patterns of class *i*. $n_{ii}$ is the number of patterns of class *i* that were classified correctly. With the above, we can define the sensitivity performance measure for class *i* as follows

(1) $$Sensitivity_{i}=\frac{n_{ii}}{N_{i}}$$

A second performance measure is defined for class *i*. To do this, we take any class *j* that is different from class *i*. That is 1≤j≤k and j≠i. $N_{j}$ is the total of patterns that belong to class *j* and $n_{ji}$ is the number of patterns that are classified as class *j*, this being an error because they belong to class *i*. This misclassification can be defined as

(2) $$N_{i}-n_{ii}$$

The total of patterns that are correctly classified as not belonging to class *i* can be defined as

(3) $$\sum_{j=1,j\neq i}^{k}\left(N_{j}-n_{ji}\right)$$

It is ensured that the total of patterns that do not belong to class *i* is calculated as follows

(4) $$\left(\sum_{j=1}^{k}N_{j}\right)-N_{i}=\sum_{j=1,j\neq i}^{k}\left(N_{j}\right)$$

Based on Equations (3) and (4), the performance measure specificity for class *i* is defined as

(5) $$Specificity_{i}=\frac{\sum_{j=1,j\neq i}^{k}\left(N_{j}-n_{ji}\right)}{\sum_{j=1,j\neq i}^{k}\left(N_{j}\right)}$$

Balanced accuracy for class *i* is defined as

(6) $$BalancedAccurracy_{i}=\frac{Sensitivity_{i}+Specificity_{i}}{2}$$

(7) $$ROCarea_{i}=\frac{Sensitivity_{i}}{1-Specificity_{i}}$$

**Figure 3:**
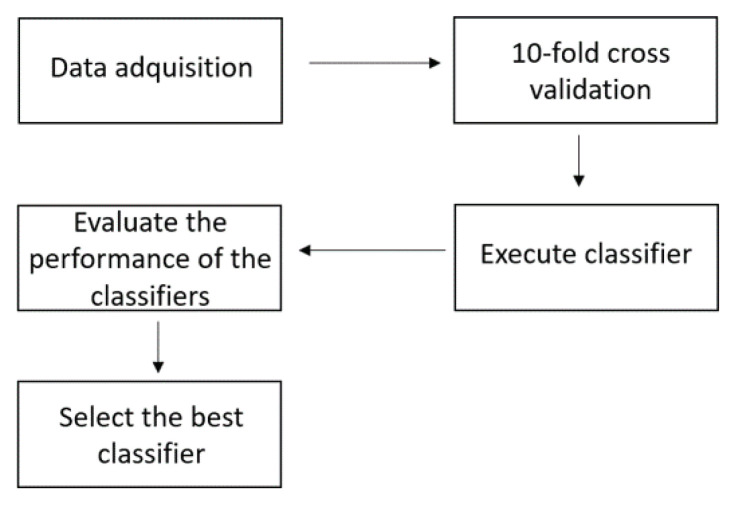
Selection process of the best classifier.

Figure 3 shows the process used for performing to select the best classifier. Data acquired by the EOG was stored in a .csv file and consists of the x,y coordinates and the class to which the eye movement belongs. The data is partitioned according to the 10-fold cross validation method and the partitions are presented to the classifiers. The performance of the classifiers are evaluated by the ROC area metric to select the most appropriate classifier for the EOG system.

#### 2.1.8. Ranking Metric Results

Characteristics of each classifier were analized by eye movement. It is divided into positive and negative action potentials; for the first case we have the movements to the right and up, while for the second case the movements down and to the left are recorded; these patterns are identified by a data vector called *p* that has the waveform of each EOG channel; each eye movement is assigned an integer value that describes the class to which it belongs. The results obtained of each classifier for each eye movement are presented.

From the results of Table 1, the multilayer perceptron classifier is chosen due to it being an average value of sensitivity, precision, specificity and balanced accuracy of all the analyzed algorithms. It best adapts to the following requirements of the developed HMI:

- A newly created dataset of each individual;
- The model resulting from the classifier implemented in an embedded system with memory characteristics lower than those of a personal computer;
- To determine the most appropriate classifier, the computational cost and the time required for each classifier were considered. Since these are higher the more accurate the classifier is, the multilayer perceptron classifier represents a balance between computational resources and accuracy.

The configuration of the MLP was: Adam optimizer, W synaptic weights and b polarization values, with 3000 epochs, four hidden nodes and two layers. W synaptic weights and b polarization values are found in the results section.

MLP classifier was implemented in Python and the code is shown in Algorithm 1.

### 2.2. EOG Signal

**Figure 4:**
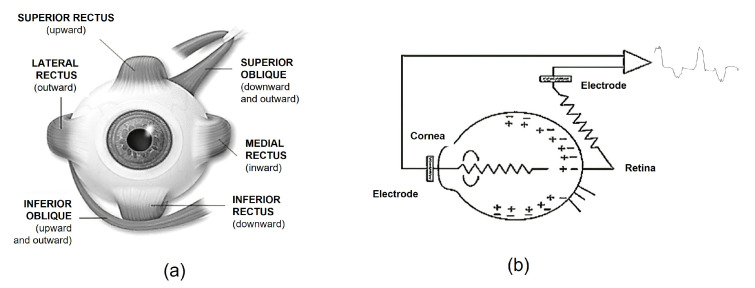
(**a**) Muscles involved in the orientation of the eyeball. Six muscles (per eye) are responsible for generating all movements of the eyes in their bony orbits: Lateral rectus, medial rectus, superior rectus, inferior rectus, superior oblique and inferior oblique. (**b**) Model of the ocular dipole of the EOG registry. Measurement of action potential differential between cornea and retina.

The human eye is the anatomical organ that makes the vision process possible. It is a uniform organ located on both sides of the sagittal plane, within the bony cavity of the orbit. The eyeball is set in motion by the oculomotor muscles that support it (Figure 4a). The EOG measures the action potential differential between the cornea and the retina, called eye dipole, which is generated with each eye movement. A change in the orientation of the dipole reflects a change in the amplitude and polarity of the EOG signal, as seen in Figure 4b, from which the movement of the eyeball can be determined [21].

**Figure 5:**
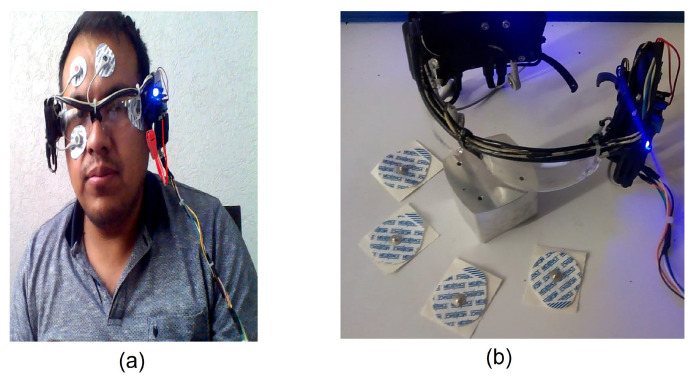
(**a**) Correct placement of the electrodes near the eyes, in the ear lobe and on the forehead. Six electrodes are used for horizontal and vertical movement signals. (**b**) Glasses designed to acquire the EOG signal, the system allows the correct placement of the electrodes on the face for signal acquisition.

Six silver/silver chloride (Ag/AgCl) electrodes are used for obtaining two channels recording horizontal and vertical eye movements. Two pairs are positioned close to the eyes, one on the earlobe and the other on the forehead, as shown in Figure 5a. EOG signals have amplitudes of 5 μV to 20 μV per degree of displacement, with a bandwidth of 0 to 50 Hz [13]. Eye movements useful for generating commands are saccadic movements, rapid movements of the eyes between two fixation points, which can be performed voluntarily or in response to visual stimulation. They reach a maximum displacement of ±45°, which corresponds to the ends of the eye position [21].
**Algorithm 1:** MLP algorithm implemented for the EOG.1P←Input_vector2T←Output_vector3 4scaler←StandardScaler().fit(P)5P←scaler.transform(P)6 7one_hot_labels=to_categorical(T,num_classes←5)8P_train,P_test,T_train,T_test←train_test_split(P,one_hot_labels,test_size←0.20,random_state←42)9 10epochs←300011hiddenNodes←412 13model←Sequential()14$model.add(Dense\left(hiddenNodes,activation\leftarrow relu,input_{d}im\leftarrow 3\right))$15$model.add(Dense\left(5,activation\leftarrow^{\prime }softmax^{\prime }\right))$16 17model.summary()18 19$loss\leftarrow categorical_{c}rossentropy$20optimizer←tf.keras.optimizers.Adam()21 22$model.compile(loss\leftarrow loss,optimizer\leftarrow optimizer,metrics\leftarrow {[}^{\prime }accuracy^{\prime }])$23$history\leftarrow model.fit(P\_train,T\_train,epochs\leftarrow epochs,verbose\leftarrow 1,validation_{s}plit\leftarrow 0.1)$24test_loss,test_acc←model.evaluate(P_test,T_test,verbose←1)25 26weights(model.layers,3)27scaling(scaler,3)28layers(model.layers)

### 2.3. Design of the HMI EOG

An HMI using EOG must be ergonomic and non-invasive [2]. For this reason, a system was developed using glasses as an optical instrument, which allows the correct placement of the electrodes on the face and embedding of the cards designed for the signal acquisition, as indicated in Figure 5b. Furthermore, users are willing to use them without fear.

**Figure 6:**
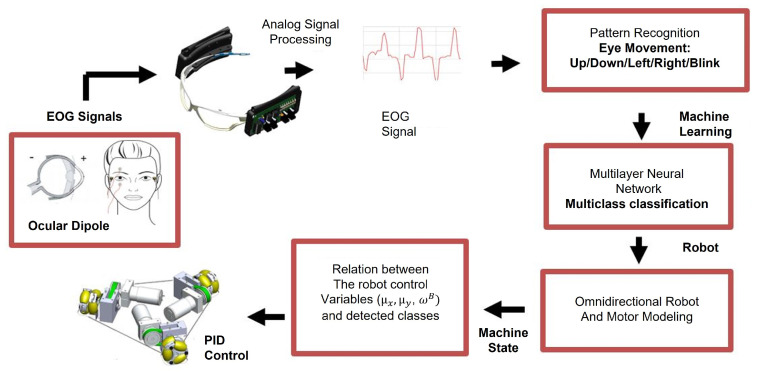
HMI structure. It includes: EOG signal acquisition, signal processing, pattern recognition, multiclass classification, relationship between robot control variables and detected classes, PID control and ominidirectional robot movement.

The proposed EOG-based HMI architecture is presented in Figure 6, in this section the signal processing modules are described.

#### 2.3.1. Analog Signal Processing

To ensure proper acquisition of the EOG signal, this module must meet the following characteristics:

- Use operational amplifiers with a high Circuit Mode Ratio Rejection (CMRR);
- Use a reference terminal connected to the forehead to decrease inductive noise and DC component;
- The electrodes must be fixed to the skin. The best location is in the periphery of the eye, in places with a greater bone proportion.

**Figure 7:**
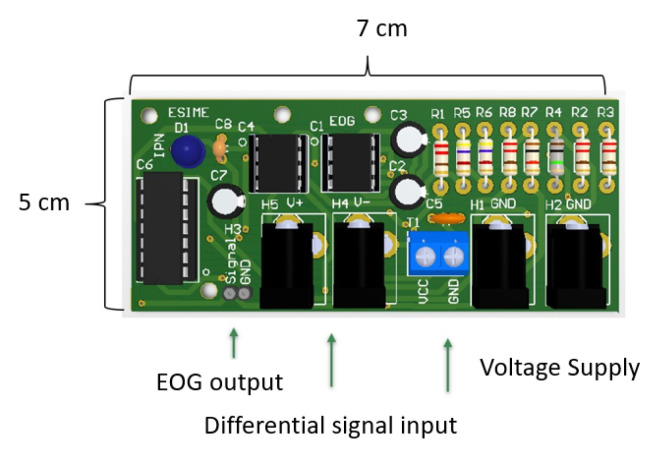
Portable EOG acquisition card. It was designed for analog processing including amplification, isolation and filtering modules.

Consider the floating ground system for the elimination of muscle noise by means of an electrode connected to the earlobe. A portable EOG acquisition card was designed for analog processing that includes modules of amplification, isolation and filtering, which are described in this section. In addition, the designed board includes a noise-reducing ground layer and hospital-grade cables for connection to decrease inductive interference, see Figure 7.

**Figure 8:**
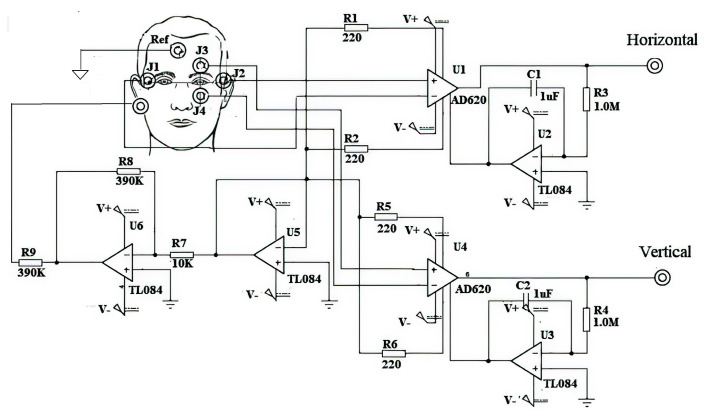
Amplification module and DC elimination module. It is implemented using an AD620 Instrumentation Amplifier with a CMRR greater than 100 dB, to remove the DC level an Integrator circuit is used for feedback concerning the EOG signal at the reference terminal of the AD62.

Amplification and DC elimination module. A preamplification stage was designed to obtain the differential signal and amplify it with a gain of 100, as the acquired EOG signal was in microvolts. An amplification system with a gain of 50 is connected to reach the voltage level necessary to sample the signal. It is implemented using an AD620 Instrumentation Amplifier (Analog Devices, Norwood, MA, USA) with a CMRR greater than 100 dB. To remove the DC level an Integrator circuit is used for feedback concerning the EOG signal at the reference terminal of the AD620, see Figure 8. It acts as a high pass filter preventing instrumentation amplifiers are saturated.

The muscle signal is considered as noise and it does not allow to obtain a good interpretation of the EOG signal. To eliminate it, the output of the common-mode circuit of the AD602 amplifier is connected to the earlobe through an electrode so as to return noise of the muscle signal at the input of the amplifier, thus the AD620 subtracting the noise signal of the EOG signal affected by noise. Additionally, the electrode placed on the the user’s forehead is connected to the isolated ground of the circuit. Through these connections the D.C. component, generated by involuntary movements and poor electrode connection, is eliminated.

**Figure 9:**
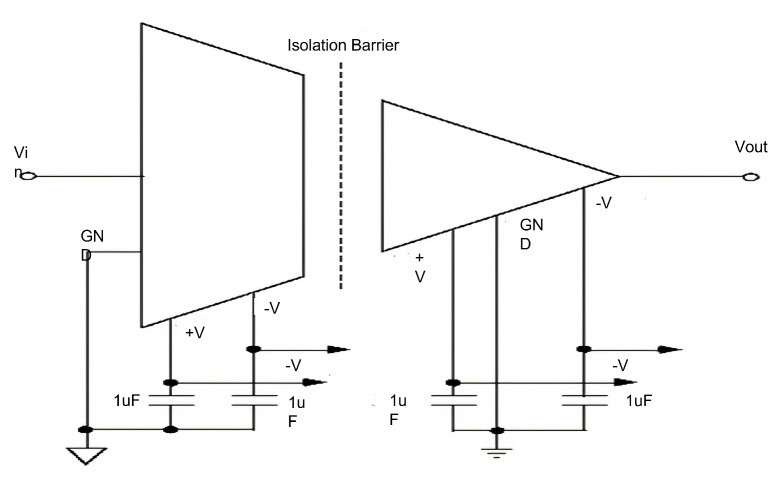
Isolation module using the IC ISO122 that generates a modulation–demodulation process using electrolytic capacitors of 1 μF.

Isolation module. For the user’s safety, a physical capacitive electrical isolation was implemented between the user and the measurement system, using the IC ISO122 (Texas Instruments, Dallas, TX, USA) that generates a modulation–demodulation process using electrolytic capacitors of 1 μF, see Figure 9.

**Figure 10:**
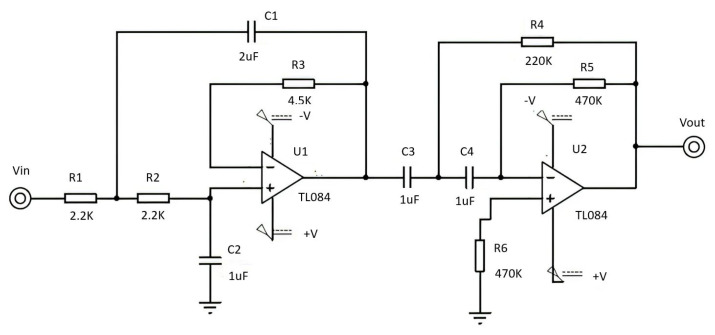
Second order filters in Butterworth configuration at 40 dB/decade. It helps to remove frequency components that are outside the bandwidth of the EOG signal, 0.5 Hz to 50 Hz.

Analog filters. To remove frequency components that are outside the bandwidth of the EOG signal, 0.5 Hz to 50 Hz, a second order band-pass filter was designed in a Butterworth configuration, with a unity gain of 40 dB per decade, using TL084 high impedance op amps, precision resistors and tantalum capacitors, Figure 10.

#### 2.3.2. Digital Signal Processing

**Figure 11:**
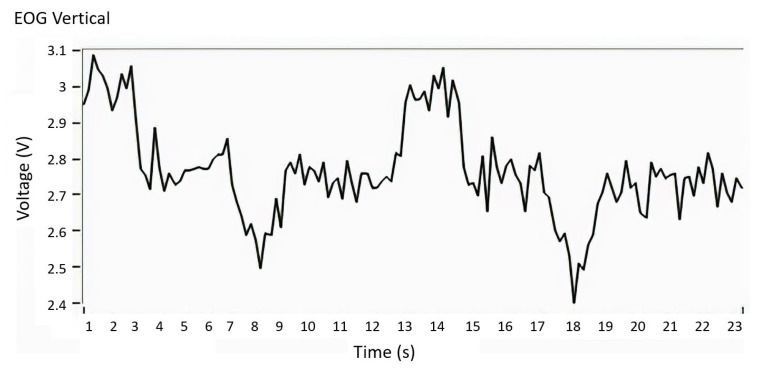
EOG signal with induced noise. Acquisition of EOG signal with induced noise as interference of unknown frequency.

The output of the acquisition stage of each EOG channel was connected to the differential voltage input of a DAQ6009 acquisition card that communicates with a PC through a USB port at a data transfer rate of 500 Hz sufficient for EOG signal sampling rate. When the EOG signal is acquired, induced noise appears as interference of unknown frequency, see Figure 11. The objective of this stage is to design a digital notch filter, to eliminate unknown noise frequencies, using Fast Fourier Transform (FFT). The EOG signal is sampled by implementing a convolution with a Dirac delta pulse train as a function of time, where x[n] is a signal represented in the Fourier exponential series, with $a_{k}$ as the energy amplitude of the signal.

(8) $$x\left[n\right]=\sum_{k=N}a_{k}e^{j\frac{e\pi }{N}kn}=a_{0}e^{j\frac{e\pi }{N}0n}+a_{1}e^{j\frac{e\pi }{N}1n}+\ldots +a_{0}e^{j\frac{e\pi }{N}\left(N-1\right)n}$$

Frequency spectrum analysis is performed by applying the Fourier transform to the discrete signal x[n], resulting in a delta function train in frequency $X(e^{jw})$, whose amplitude is determined by the coefficients $a_{k}$. Using Equation (9), the discrete signal is transformed to the Fourier exponential form; the frequency spectrum determines the energy components of the EOG signal.

(9) $$X\left[e^{jw}\right]=\sum_{k=-\infty }^{\infty }a_{k}2\pi \delta \left(w-\frac{2\pi }{N}k\right)$$

**Figure 12:**
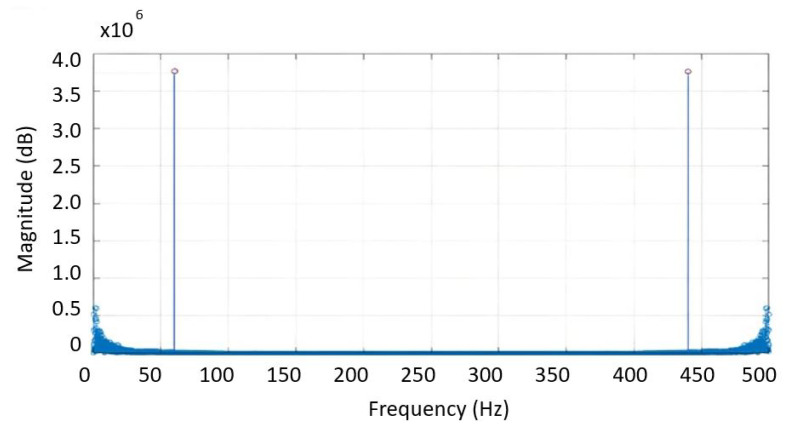
Frequency Spectrum of the EOG signal from FFT; according to this the cut-off frequency for the design of the digital filter is 60 Hz.

In Figure 12, the frequency component that provides the most energy is the 60 Hz signal; this data accurately provides the frequency of the unknown induced noise and the cut-off frequency for the design of a digital notch filter; the transfer function is the Equation (10):

(10) $$H\left(z\right)=\frac{Y(z)}{X(z)}=\frac{\left(z-e^{j\Omega }\right)\left(z-e^{-j\Omega }\right)}{\left(z-rie^{j\Omega }\right)\left(z-rie^{-j\Omega }\right)},$$

where Ω is the digital angular frequency, which is related to the analog angular frequency *w*,

(11) Ω=Tw

with *T* as the sampling period, *r* is the value within the unit radius circle in the *z* plane, where the desired complex-conjugate poles must be located for the design of the filter, whose relation to the filter bandwidth (BW) is Equation (12),

(12) $$r\approx 1-\frac{BW}{f_{s}}\pi$$

To calculate the filter transfer function, a pair of complex-conjugated zeros are placed in the unit radius circle in the *z* plane, corresponding to the 60 Hz frequency. The coordinates in the complex plane where these zeros will be located, are calculated from the digital frequency, Equation (11), using the sampling rate of the acquisition, 500 Hz and the cutoff frequency for the 60 Hz filter.

(13) $$\Omega =Tw=\pm 360^{\circ }\left(\frac{60}{500}\right)=\pm 43{}^{\circ}=\frac{43}{180}\pi$$

**Figure 13:**
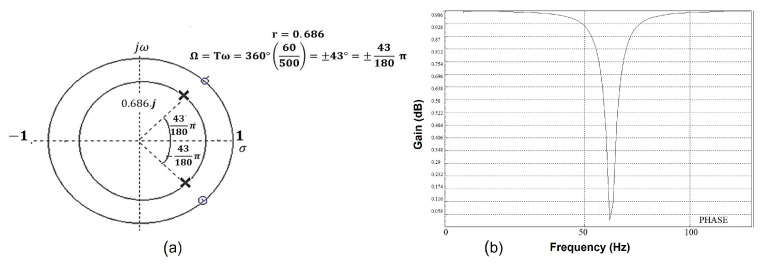
(**a**) Map of poles and zeros of the digital filter. The location in the colplex plane *z* of poles and zeros for the filter design. (**b**) Notch digital filter phase diagram. Frequency response of the designed filter.

To design the band reject filter, a pair of conjugated complex poles are placed at an angle given by Ω with a radius *r*, calculated from Equation (12) with design specifications BW = 50 Hz and $f_{s}$ = 500 Hz. Figure 13a shows the location in the complex plane *z* of the poles and zeros used for the filter design; Figure 13b shows the frequency response of the designed filter.

(14) $$r\approx 1-\frac{BW}{f_{s}}\pi =1-\frac{50}{500}\pi =0.686$$

The corresponding transfer function for the notch filter is presented in

(15) $$\begin{array}{c} H\left(z\right)=\frac{Y(z)}{X(z)}=\frac{\left(z-e^{j\frac{43\pi }{180}}\right)\left(z-e^{-j\frac{43\pi }{180}}\right)}{\left(z-0.686ie^{j\frac{43\pi }{180}}\right)\left(z-0.686ie^{-j\frac{43\pi }{180}}\right)}\\ H\left(z\right)=\frac{z^{2}-1.4627z+1}{z^{2}-1.00342z+0.470596} \end{array}$$

**Figure 14:**
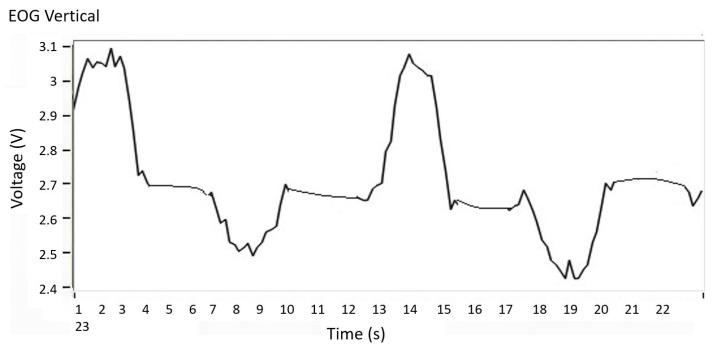
Filtered EOG signal. EOG signal free of induced noise.

By performing a digital convolution between the EOG signal and the filter transfer Equation (15), a signal is obtained without the induced noise. With the inverse transform Z, the discrete signal changes to a continuous signal, the result is presented in Figure 14, where the EOG signal free of induced noise is observed.

### 2.4. Classification of the EOG Signal by Multilayer Perceptron

In this section, the implementation of an intelligent system for the classification of the movement of the eyeball acquired through EOG is presented. The first stage consists of data normalization since the EOG thresholds have different scales and intervals; the implementation of this technique is described in Equation (16). Where $p^{2}$ represents the dataset of the EOG signal through a vector with two channels, this will be the input of the neural network; the mean of the data is subtracted with a standard deviation equal to 1 to minimize the learning difficulty of the neural network.

(16) $$p^{2}=\frac{p-p^{mean}}{\sqrt{p^{var}}}=\frac{p-p^{mean}}{p^{std}}$$

**Figure 15:**
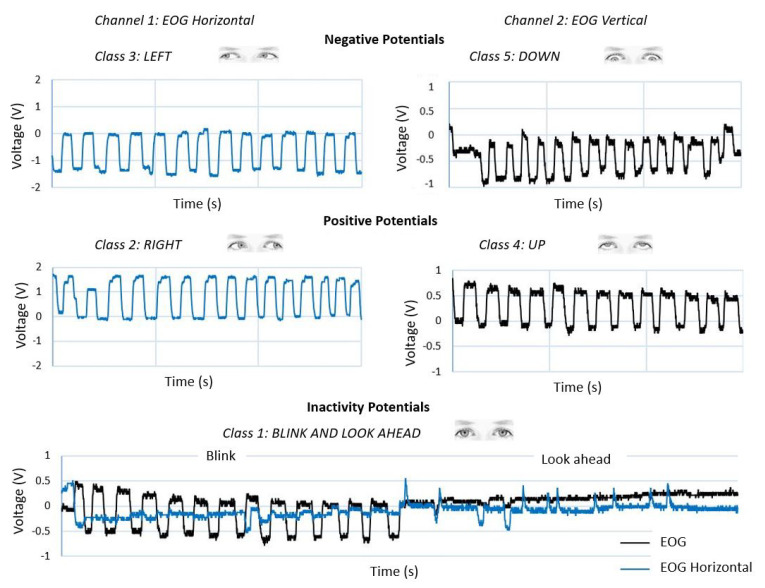
Graphical representation of the dataset. Horizontal and vertical EOG waveforms and the eye movement they represent; class 1: Blink and look ahead; class 2: Right; class 3: Left; class 4: Up; class 5: Down.

To perform the identification of patterns in the EOG signal through two channels, they are divided into negative action potentials (left/down), positive action potentials (right/up) and inactivity potentials (blinking and involuntary movements when looking straight ahead). Each of these classes of the EOG signal is labeled by an integer. This type of encoding is appropriate if there is an order relationship between the categories; this type of data is known as ordinal. Figure 15 shows the waveform of each of the EOG channels and the eye movement it represents; also, the detection of blinking in both EOG channels (horizontal/vertical) is added to the training dataset in order to prevent involuntary movements being recognized as control commands.

**Figure 16:**
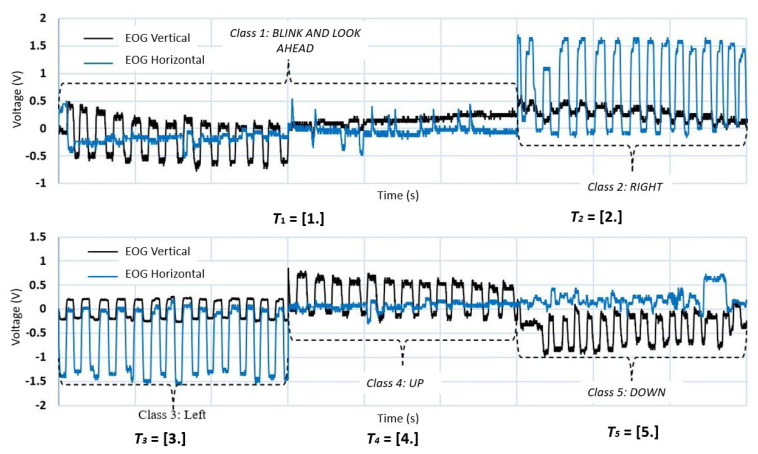
Graphical representation of the dataset (input vector $p^{2}$). Input vector $p^{2}$ represents the class 1: Blink and look ahead; class 2: Right; class 3: Left; class 4: Up and class 5: Down, and output vector $T^{2}$ labeling stored by one-hot encoding of each class.

**Figure 17:**
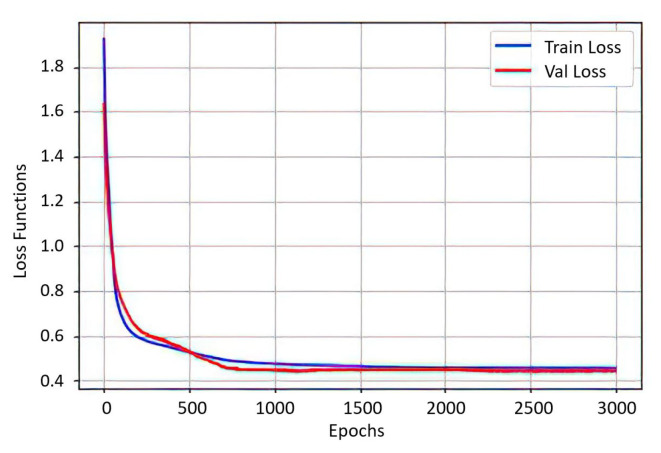
Graph of the trend of the neural network accuracy with new data (train loss) and the trend of the loss function (val loss). Relation of the precision of the neural network after 3000 epochs.

Figure 16 shows the labeling of each class for the two EOG channels and Algorithm 2 shows the pseudocode for the implementation of the neural network in Python; in Figure 17 there is an association between the precision of the neural network with new data (train loss) and the value of the loss function (val loss) after 3000 epochs; both graphs have a tendency to zero as the training progresses, presenting a correct operation of the optimizer. The training was carried out by assigning to each sample the value of a constant stored in the vector (T]); this vector is the desired result for each class of the same size as the input vector (*p*); through this labeling, supervised training of the MLP is enabled.

Values obtained from the synaptic weights W and the polarization vectors b of the two neurons, after 3000 epochs:

$$W_{1}=\left[4\right]\left[2\right]=\left[\begin{array}{cc} -0.325 & 0.128\\ 0.372 & 0.299\\ 0.077 & 0.470\\ -0.084 & -0.792 \end{array}\right]$$

$$W_{2}=\left[5\right]\left[4\right]=\left[\begin{array}{cccc} -0.567 & -0.149 & -0.361 & -1.479\\ 0.226 & 0.164 & -0.345 & 0.027\\ 0.034 & 0.352 & -0.510 & 0.113\\ -0.041 & -0.056 & 0.143 & 0.139\\ -0.267 & -1.245 & 0.346 & 0.325 \end{array}\right]$$

$$b_{1}=\left[\begin{array}{cccc} -0.057 & -0.094 & 0.064 & 0.138 \end{array}\right]$$

$$b_{2}=\left[\begin{array}{ccccc} 0.679 & -0.051 & 0.100 & -0.482 & -0.355 \end{array}\right]$$

#### 2.4.1. Omnidirectional Robot

**Figure 18:**
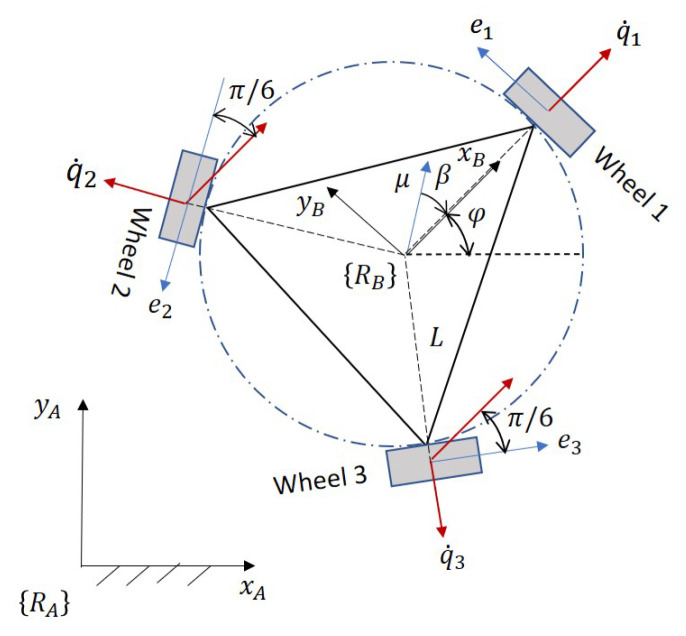
Graphical representation of the omnidirectional three-wheeled robot to obtain its forward kinematics. The robot can rotate on its own axis, rotate on the vertical axis and slide in all directions.

The system to be controlled is an omnidirectional three-wheeled robot that can rotate on its own axis, rotate on the vertical axis and slide in all directions. The three degrees of freedom that the robot has are defined by the variables $\mu_{y}$ which represents the linear speed that moves the robot in the right and left directions, the variable $\mu_{x}$ represents the linear speed that moves the robot in up and down directions, while the variable *w* represents the angular velocity of the robot, as indicated in Figure 18. The kinematic model must consider the characteristics of the omnidirectional robot, with a Swedish three-wheel structure and a space of 120° between them, considering the contribution of each of the wheels to the robot’s speeds $\mu_{y}$ and $\mu_{x}$, that is, the radius of each wheel times the angular velocity $\left(R_{1}{\dot{q}}_{1},R_{2}{\dot{q}}_{2},R_{3}{\dot{q}}_{3}\right)$, results in the individual linear velocity. The vector sum of each of these speeds is the robot’s center speed.

**Algorithm 2:** Neural network pseudocode.

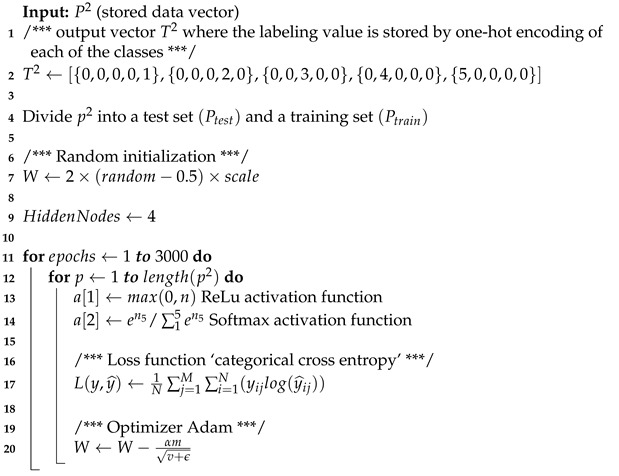



Two coordinate systems are defined (Figure 18), the fixed coordinate system $\left\{R_{A}\right\}$ and the coordinate system at the point of interest $\left\{R_{B}\right\}$ in the robot; the $x_{B}$ axis is perpendicular to wheel 1; between the $x_{A}$ and $x_{B}$ axes the robot orientation angle φ is defined; the orientation of wheels 2 and 3 are measured with respect to the $x_{B}$ axis; the planes of each of the wheels are determined by the axes $e_{1}$, $e_{2}$ and $e_{3}$.

In the model of Figure 18 the angular speed ${\dot{\varphi }}^{A}$ calculated in the coordinate space $\left\{R_{A}\right\}$ is equal to $w^{B}$ in the Cartesian space $\left\{R_{B}\right\}$; *L* represents the distance between the center of the robot and the wheel axis. The angular velocity of each wheel is expressed by the variables ${\dot{q}}_{1}$, ${\dot{q}}_{2}$ and ${\dot{q}}_{3}$; the speed of wheel 1 in the function of the robot speed is determined in Equation (17).

(17) $$R{\dot{q}}_{1}=\sum \mu_{e1}=-\mu_{x}\sin\varphi +\mu_{y}\cos\varphi +Lw^{B}$$

The speed of wheel 2 in the function of the robot speed is determined in Equation (18).

(18) $$R{\dot{q}}_{2}=\sum \mu_{e2}=-\mu_{x}\cos\left(\frac{\pi }{6}+\varphi \right)-\mu_{y}\sin\left(\frac{\pi }{6}+\varphi \right)+Lw^{B}$$

The speed of wheel 3 in the function of the robot speed is determined in Equation (19).

(19) $$R{\dot{q}}_{3}=\sum \mu_{e3}=\mu_{x}\cos\left(\varphi -\frac{\pi }{6}\right)+\mu_{y}\sin\left(\varphi -\frac{\pi }{6}\right)+Lw^{B}$$

Equations (17)–(19) give the inverse kinematics and the Jacobian matrix *J* (Equation (20)) to convert the linear velocity (expressed in the fixed coordinate system $\left\{R_{A}\right\}$ and the angular velocity of the robot) to the angular velocities required in each wheel to track a trajectory.

(20) $$\left[\begin{array}{c} {\dot{q}}_{1}\\ {\dot{q}}_{2}\\ {\dot{q}}_{3} \end{array}\right]=\frac{1}{R}\left[\begin{array}{ccc} -\sin\varphi & \cos\varphi & L\\ -\cos(\frac{\pi }{6}+\varphi ) & \sin(\frac{\pi }{6}+\varphi ) & L\\ \cos(\varphi -\frac{\pi }{6}) & \sin(\varphi -\frac{\pi }{6}) & L \end{array}\right]\left[\begin{array}{c} \mu_{x}\\ \mu_{y}\\ w^{B} \end{array}\right]$$

The Jacobian matrix *J* is inverted to obtain the forward kinematics model. The inverse Jacobian matrix $J^{-1}$ gets the robot’s speed in terms of the fixed coordinate space $\left\{R_{A}\right\}$ and the angular velocity of the robot as a function of the angular velocities of each wheel, expressed in Equation (21).

(21) $$\left[\begin{array}{c} \mu_{x}\\ \mu_{y}\\ w^{B} \end{array}\right]=\frac{R}{3}\left[\begin{array}{ccc} -2\sin\varphi & \sin\varphi -\sqrt{3}\cos\varphi & \sin\varphi +\sqrt{3}\cos\varphi \\ 2\cos\varphi & -\sqrt{3}\sin\varphi -\cos\varphi & \sqrt{3}\sin\varphi -\cos\varphi \\ \frac{1}{L} & \frac{1}{L} & \frac{1}{L} \end{array}\right]\left[\begin{array}{c} {\dot{q}}_{1}\\ {\dot{q}}_{2}\\ {\dot{q}}_{3} \end{array}\right]$$

To model the angular velocity ${\dot{q}}_{i}$ of the motors, a first order system with delay is implemented because the motor takes time to respond; this model is represented in the frequency domain and in the time domain (Equation (22)).

(22) $$\begin{array}{c} G\left(s\right)=\frac{Ke^{-Hs}}{\tau s}+1\\ p_{v}\left(t\right)=\left\{\begin{array}{cc} 0 & 0\leq t<H\\ K\left(1-e^{-(t-H)/\tau }\right)C_{v}\left(t\right) & t\geq H \end{array}\right. \end{array}$$

where *K* is the gain of the open-loop process, τ is the open-loop time constant and *H* is the delay.

**Figure 19:**
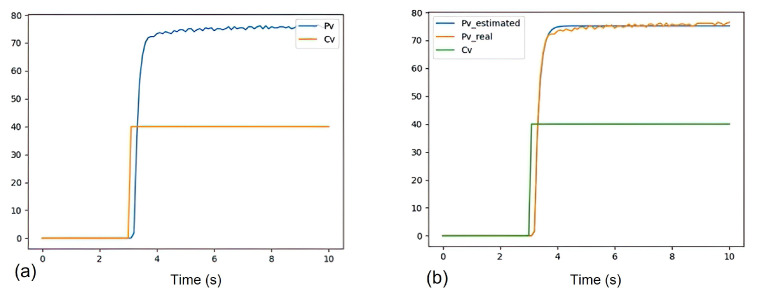
Chihai CHR-GM25 double quadrature motor from 140 RPM at 12 V within 10 s response. (**a**) Response of the motor to a step function (process variable $v_{p}$). (**b**) Approximation of $v_{p}$ by PSO.

To determine the model parameters, the variables *p_v_* and *c_v_* are defined as the process variable (motor response) and the control variable (step function), respectively; the response of the open-loop system is obtained through an input process that will be the unit step function; at the output we will have the radians over the second at which it rotates; Figure 19 shows the response of a Chihai CHR-GM25 double quadrature motor from 140 RPM at 12 V within 10 s. Subsequently, Particle Swarm Optimization (PSO) [52] is implemented, which obtains the approximation of the process variable $\left(p_{v_{estimated}}\right)$, resulting in each of the model parameters with a value of *K* = 1.8795, τ = 0.1523 and *H* = 0.0967.

The control implemented is a PID with lambda tuning since it ensures robustness, stability and non-oscillatory response; in [53], the advantage of this method over some other tuning methods is explained (Ziegler and Nichols and Haalman); in Equation (23) the standard PID control formula is indicated to tune the controller using the lambda method; the value of λ=3τ and the values of the gains $K_{p}$ (proportional gain), $T_{i}$ (integral time) and $T_{d}$ (derivative time ) of the PID controller are determined by substituting the values of the model parameters.

(23) $$\begin{array}{c} U\left(s\right)=K_{p}\left(1+\frac{1}{T_{i}s}+T_{d}s\right)E\left(s\right)\\ K_{p}=\frac{\tau }{K(H+\lambda )}=0.1463,\\ t_{i}=\tau =0.1523,\\ T_{d}=\frac{H}{2}=0.048 \end{array}$$

**Figure 20:**
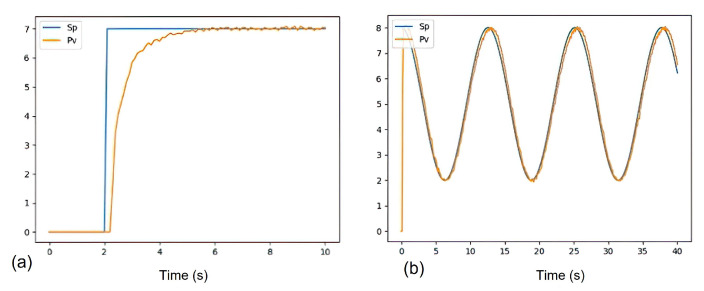
The response of the controller is tested with a step function and the follow-up of the trajectory as a cosine function. (**a**) Response of the PID control with a step function. (**b**) Response of the PID control to track a trajectory represented by the cosine function.

The response of the controller is tested with a step function and the follow-up of the trajectory as a cosine function. Figure 20 indicates the correct follow-up of the trajectory for a desired angular velocity established as $s_{p}$ (set point).

#### 2.4.2. State Machine

**Figure 21:**
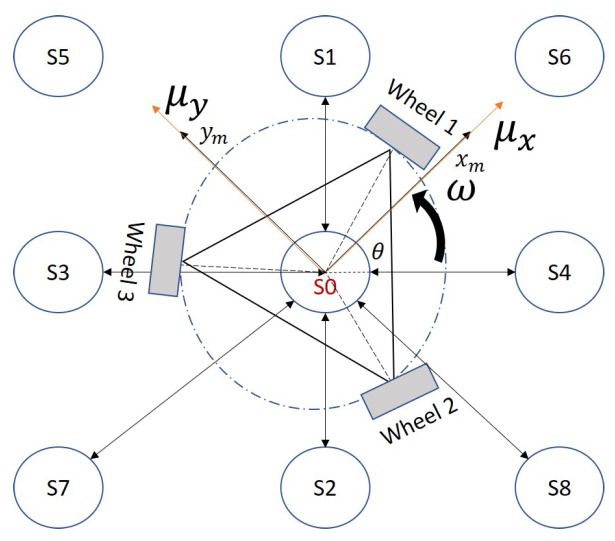
Mealy-type state machine for motion control of an omnidirectional robot; nine states are implemented for the control of a Mealy type machine. S0 to S4 for EOG class from 1 to 5 and S5 to S8 for combined and sequential linear movements.

Table 2 indicates the digital representation of each of these states and the position relationship it represents. The PID control algorithm is implemented in each of the motors to reach the reference values determined by the variables $\mu_{x}$, $\mu_{y}$ and $w^{B}$; by means of the inverse kinematics expressed in Equation (21), the the speeds ${\dot{q}}_{1}$, ${\dot{q}}_{2}$ and ${\dot{q}}_{3}$ are obtained. These values are described in Table 3 for each state of the machine. Nine states are implemented for the control of a Mealy type machine as shown in Figure 21. Through an established acquisition period, the corresponding class is detected according to the output of the neural network; the result is stored in a data vector and the new result is compared with the previous one; when there is a change in the transition, the combined and sequential movements are activated for the rotational and diagonal trajectories. In digital circuits and machine learning, one-hot is a group of bits among which the allowed combinations of values are only those with a single high bit (1) and all others low (0), one-hot encoding is implemented to relate each state of the machine and each class resulting from multiclass classification.

## 3. Results and Discussion

To evaluate the operation of the HMI, tests were developed in digital evaluation systems and simulations. First, the response of the EOG acquisition system to interference was evaluated experimentally. Later, by means of the graphic interface, simulation tests were performed to evaluate the performance of the classifier.

### 3.1. EOG Acquisition System Evaluation

**Figure 22:**
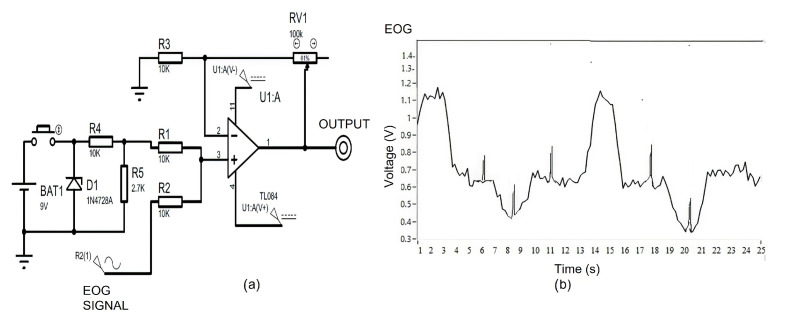
Experimental impulse function added to the input of the EOG acquisition system to evaluate the performance of the HMI system against disturbances. (**a**) System to test interference elimination. (**b**) Signal obtained with perturbation.

The environment affects the quality of the EOG signal, so a notch filter that can be calibrated in real time was designed; the cutoff frequency can be modifies according to the frequency of the detected noise, resulting in an EOG signal free of interference. Tests were performed in different work environments and 97.3% efficiency of the filtering system was obtained. To evaluate the performance of the HMI system against disturbances, such as a user blink, an impulse function was experimentally added to the input of the EOG acquisition system by means of the circuit of Figure 22a. The impulse function was modeled as a button that connects a Zener diode, which acts as a voltage source at the input of the op amp that has an adder configuration.

The signal obtained is seen in Figure 22b; the disturbance does not affect the classifier because the experimental tests determined that, even with this induced noise, the neuronal network model is capable of classifying the movement according to the class that corresponds to it.

#### Virtual Test

**Figure 23:**
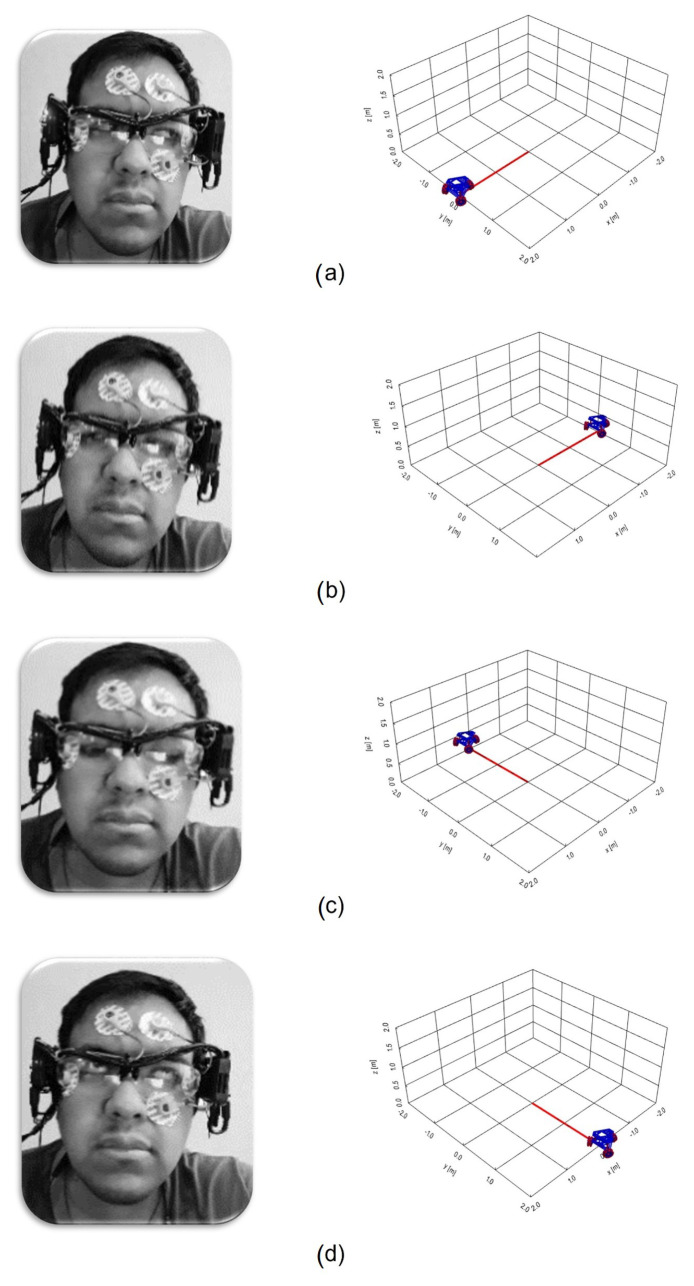
Virtual robot movements when controlled by the rotation of the eyeball. (**a**) Eye movement looking up and tracking the robot’s trajectory forward. (**b**) Eye movement looking down and tracking the robot’s trajectory backwards. (**c**) Eye movement looking to the right and tracking the robot’s trajectory to the right. (**d**) Eye movement looking to the left and tracking the robot’s trajectory to the left.

The graphical interface was used as a virtual test platform. In Figure 23a–d, the different movements that the virtual robot does when controlled by the rotation of the user’s eyeball are presented; this is a prior test conducted before connecting the HMI to the physical robot and thus evaluating whether the user can control the robot virtually by making it scroll around the workspace.

**Figure 24:**
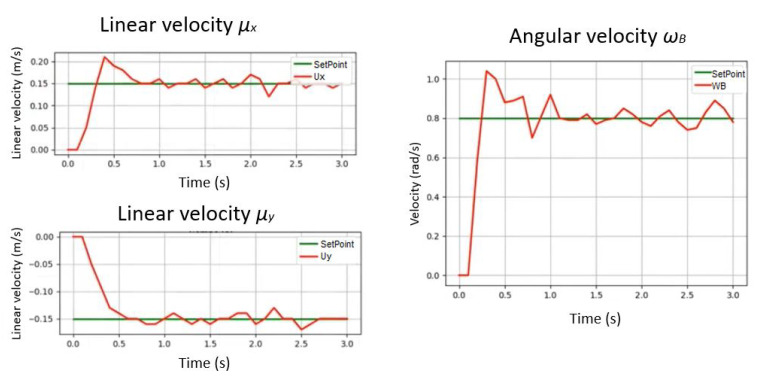
Desired values indicated in the state machine and the PID control responses for each variable $\mu_{x}$, $\mu_{y}$ and $w^{B}$.

Figure 24 indicates the monitoring of the desired values indicated in the state machine for each control variable $\mu_{x}$, $\mu_{y}$ representing the linear velocity in meters per second and $w^{B}$ representing the angular velocity in radians per second.

### 3.2. Performance Test

**Figure 25:**
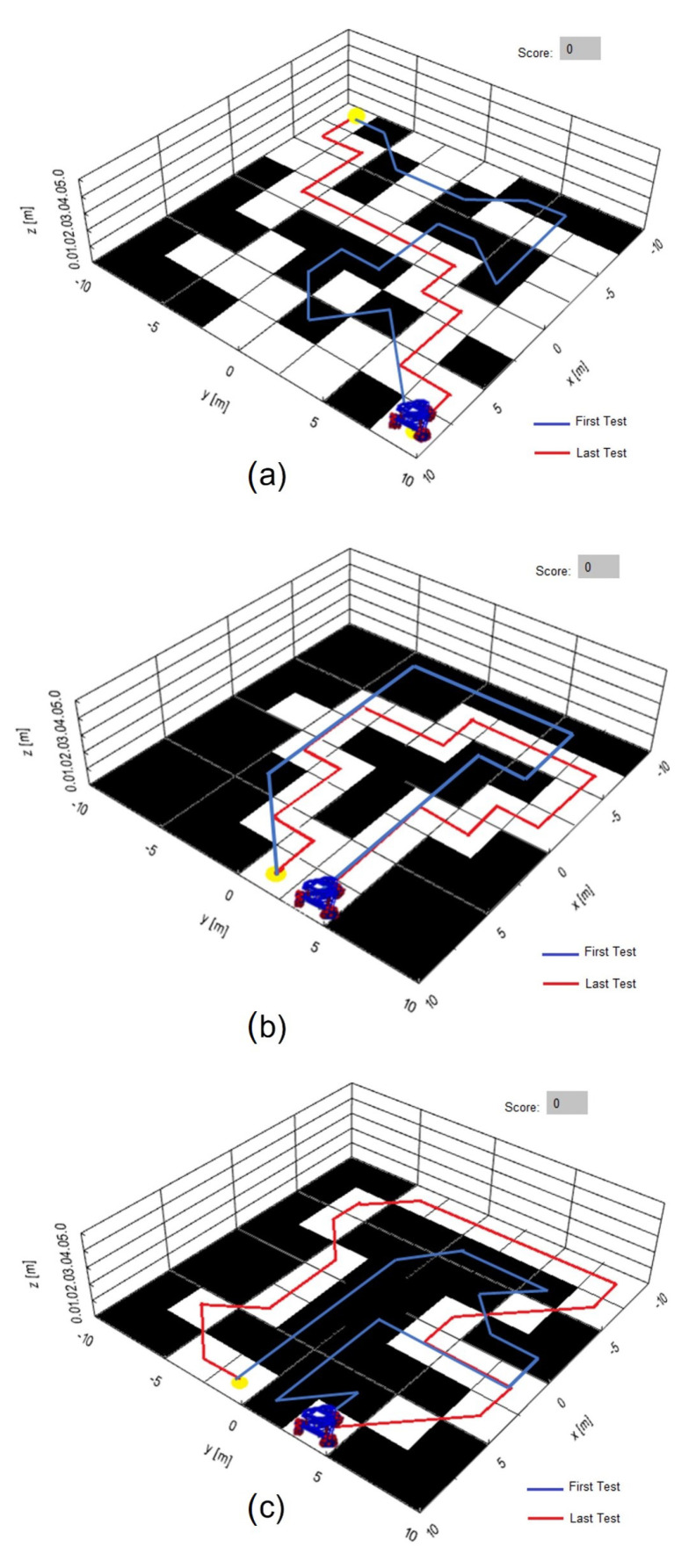
Trajectory for training boards programmed; a blue line indicates the first test carried out and a red line is the trajectory after 30 repetitions to reach zero penalties.(**a**) Test Board 1 with linear movements of the eyeball and the omnidirectional robot. (**b**) Test Board 2 with linear movements of the eyeball and the omnidirectional robot. (**c**) Test Board 3 with linear and combinational movements of the eyeball and diagonal trajectories of the omnidirectional robot.

Three game and training boards are programmed; the ability of the user to arrive from a starting point and an end point colored in yellow is evaluated; each black square on the game board corresponds to a penalty, which means there are points in the workspace where the user must not place the mobile robot; the only valid points to move the robot are the white squares. The test consists of recording the number of penalties and the time it takes for the user to place the robot on the assigned points, marking the generated trajectory in red. In Figure 25a,b, Boards 1 and 2 are shown; only linear movements are recorded. In Figure 25c, Test Board 3 is presented; linear and sequential movements are recorded, which are combinations of the eyeball to move the robot diagonally or rotationally.

The interface has the property of detecting involuntary movements such as blinking and looking forward; in Figure 25 there is also a trajectory marked in blue that indicates the first test carried out; the tests on different boards indicate that 30 repetitions is enough to reach zero penalties.

**Figure 26:**
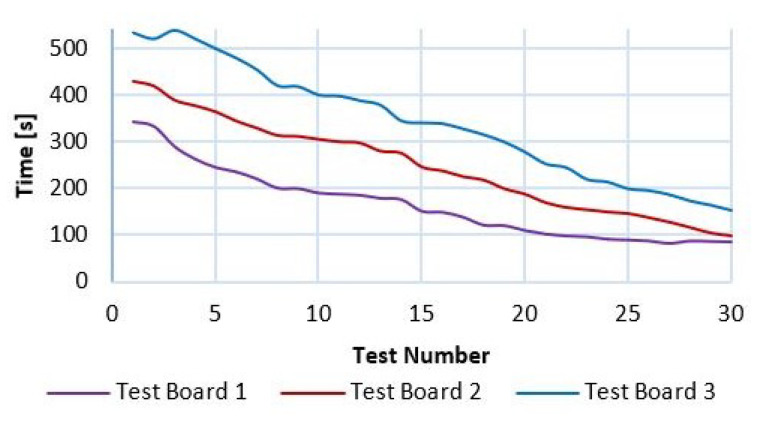
Number of tests performed and the response time recorded.

**Figure 27:**
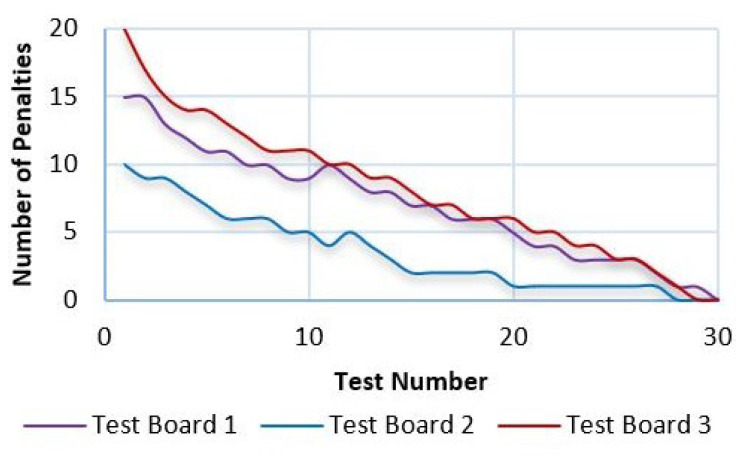
Number of tests performed and the number of penalties recorded.

In Figure 26, the trend graph of Table 4 is presented, which records the response time of each of the repetitions performed. It is observed that after 30 repetitions the time is decreased by 71.1% to perform the task on Test board 3; on Test Board 2 the time is reduced by 76.9% when executing the task and finally on Test Board 1 there is a response time reduction of 75.4%. The experiment ends after 30 repetitions since there were 0 penalties decreasing after each repetition. This result can be seen in Figure 27, which indicates the downward trend in the number of penalties recorded in Table 5. Therefore, a conclusion can be obtained where, regardless of the test board, the user has a mastery after 30 repetitions with an average of 74.5% reduction of learning time.

In the previous results, regardless of the game board, the percentage level in the reduction of the test time is maintained at 74.5% by having zero penalties; if this percentage is converted to a decimal value, it is 0.745, an approximate value to the sensitivity and precision of the MLP classifier which is 0.755; this means that there is a direct relationship between the classifier measurements and the time in the reduction of the HMI response. The reductions in the response time of the classifier when registering new data in the experiment measures the sensitivity and precision of the MLP with new data are similar to the precision and sensitivity that the classifier has with data already stored, from which it can be deduced that the model obtained from the classifier programmed in an embedded system to control a robotic device does not lose effectiveness.

The results are explained by the good performance of the EOG signal classifier. The faster the response of the HMI system, the better the acceptance of the user, fulfilling the objective that people really feel a domain and control over a mobile robot that in the future will be able to adapt to assistance systems.

It is important to evaluate user satisfaction with the HMI system and its influence on human–robot interaction. The advantage of this system is that it adapts to the individual properties of the user, allowing optimal performance in signal classification. This provides a considerable decrease in the response time of the HMI system compared to other works presented in literature. There are several studies that have shown that users lose interest with assistive devices that have a long response time, this being one of the key factors why they are rejected, making it so that the system presented in this work will have a high acceptance by the end user due to the short response time.

## 4. Conclusions

The design of an HMI system developed a new method of classifying EOG signals that allows real-time generation of trajectories in the (X, Y) plane.

The HMI works with any inexperienced user because the system adapts to personal characteristics after a short training of no more than 30 tests. For future patient trials, approval of an ethical protocol will be included. The classifier has the property of discriminating involuntary movements such as blinking and if some of these movements occur the robot does not move, which allows the user to control the robot by having a stop option.

This HMI customizes the use of assistive devices when using physiological signals, reducing training time. Due to these characteristics, this HMI could be very useful to support people with disabilities in their routine activities, to control devices such as wheelchairs, assistance robots, virtual keyboards and mice, digital pointers and home automation controls. 

## Figures and Tables

**Table 1:** Description of performance measures (sensitivity, specificity, balanced accuracy and precision) in different classifiers.

Classifier	Sensitivity	Specificity	Balanced Accuracy	Precision	ROC Area
Random Forest	0.986	0.982	0.984	0.986	0.999
Random Tree	0.986	0.982	0.984	0.986	0.996
J48	0.977	0.973	0.975	0.977	0.996
KNN-1	0.979	0.975	0.977	0.979	0.997
KNN-2	0.966	0.960	0.963	0.966	0.997
KNN-3	0.958	0.952	0.955	0.958	0.996
Logistic	0.683	0.805	0.744	0.683	0.889
Multilayer Perception	0.755	0.836	0.795	0.755	0.889
Support Vector Machine	0.669	0.853	0.761	0.669	0.882
Naive Bayes	0.714	0.849	0.782	0.714	0.905

**Table 2:** Categorization by integers (one-hot encoding).

# Class	One Hot Encoding/State Machine				
Ocular Movement	S3 (Left)	S2 (Down)	S4 (Right)	S1 (Up)	S0 (Stop)
3 (Left)	1	0	0	0	0
5 (Down)	0	1	0	0	0
2 (Right)	0	0	1	0	0
4 (Up)	0	0	0	1	0
1 (Stop)	0	0	0	0	1

**Table 3:** Description of each of the movements in the state machine.

State	Class EOG	Desired Value $\left(\mu_{x},\mu_{y},w^{B}\right)$	Desired Movement
S3	3	(−0.15 m/s, 0 m/s, 0 rad/s)	Left
S2	5	(0 m/s, −0.15 m/s, 0 rad/s)	Down
S4	2	(0.15 m/s, 0 m/s, 0 rad/s)	Right
S1	4	(0 m/s, 0.15 m/s, 0 rad/s)	Up
S0	1	(0 m/s, 0 m/s, 0 rad/s)	Stop
Combined and sequential linear movements			
S5	3, 4	(−0.15 m/s, 0.15 m/s, 0 rad/s)	Upper-Left Diagonal
S6	2, 4	(0.15 m/s, 0.15 m/s, 0 rad/s)	Upper-Right Diagonal
S7	3, 5	(−0.15 m/s, −0.15 m/s, 0 rad/s)	Lower-Left Diagonal
S8	2, 5	(0.15 m/s, −0.15 m/s, 0 rad/s)	Lower-Right Diagonal
Combined and sequential rotational movements			
4, 2, 5, 3		(0 m/s, 0 m/s, 0.8 rad/s)	Counterclockwise rotation
3, 5, 2, 4		(0 m/s, 0 m/s, −0.8 rad/s)	Clockwise rotation

**Table 4:** Summary of response time in seconds on each test board.

Test Number	Test Board 1	Test Board 2	Test Board 3
1	343.50	430.30	532.90
5	245.12	365.10	499.12
10	189.80	306.00	400.20
15	150.32	246.60	340.90
20	108.87	188.50	278.40
25	88.65	147.1	200.23
30	84.32	99.32	154.23

**Table 5:** Summary of the number of penalties.

Test Number	Test Board 1	Test Board 2	Test Board 3
1	15	10	18
5	11	7	14
10	9	5	11
15	7	2	8
20	5	1	6
25	3	1	3
30	0	0	0
